# The East Africa Infection Prevention and Control (IPC) Learning Network: An Approach to Improving IPC Competencies and Practices During the COVID-19 Pandemic, 2020–2023

**DOI:** 10.1093/cid/ciaf245

**Published:** 2025-06-24

**Authors:** Getachew Kassa, Irene Ogongo, Miriam Rabkin, Elizabeth Bancroft, Rick Mitchell, Laura Block, Cori Dennison, Elizabeth Katwesigye, Markos Paulos, Joseph Hokororo, Irungu Kamau, Carolyn Herzig

**Affiliations:** International Center for AIDS Care and Treatment Program (ICAP) at Columbia University, New York, New York, USA; International Center for AIDS Care and Treatment Program (ICAP) at Columbia University, New York, New York, USA; International Center for AIDS Care and Treatment Program (ICAP) at Columbia University, New York, New York, USA; Division of Healthcare Quality Promotion, US Centers for Disease Control and Prevention, Atlanta, Georgia, USA; International Center for AIDS Care and Treatment Program (ICAP) at Columbia University, New York, New York, USA; International Center for AIDS Care and Treatment Program (ICAP) at Columbia University, New York, New York, USA; Division of Healthcare Quality Promotion, US Centers for Disease Control and Prevention, Atlanta, Georgia, USA; Infection Prevention and Control, Uganda Ministry of Health, Kampala, Uganda; Infection Control Unit, Ethiopia Federal Ministry of Health, Addis Ababa, Ethiopia; Health Quality Assurance Unit, Tanzania Ministry of Health, Dar es Salaam, Tanzania; Division of Patient and Health Worker Safety, Kenya Ministry of Health, Nairobi, Kenya; Division of Healthcare Quality Promotion, US Centers for Disease Control and Prevention, Atlanta, Georgia, USA

**Keywords:** infection prevention and control (IPC), East Africa, COVID-19, SARS-CoV-2, healthcare workers

## Abstract

**Background:**

Outbreaks of Ebola and the COVID-19 pandemic demonstrate that healthcare workers (HCWs) are critical for resilient health systems. Interventions that improve infection prevention and control (IPC) practices are required to protect HCWs. We aimed to implement a regional IPC learning network to improve compliance with IPC standards.

**Methods:**

This project was implemented in a network of 20 tertiary care hospitals in Ethiopia, Kenya, Tanzania, and Uganda. Baseline and routine assessments of hospital IPC and IPC focal point competencies were conducted from January 2021 through June 2023 to identify gaps and measure progress. Virtual and in-person trainings were held routinely, and a collaborative quality improvement (QI) project on personal protective equipment (PPE) use was conducted. Data were analyzed to describe changes in IPC compliance and competencies.

**Results:**

Overall, hospital compliance with IPC standards improved from baseline to the final assessments across all domains assessed. IPC focal points’ occupational health competency scores increased; median scores for each competency component ranged from 2.5 to 3.5 (out of 5) at baseline and were ≥4.5 at endpoint. Eighteen hospitals completed the QI collaborative; average compliance with appropriate PPE use across hospitals increased significantly, from 65% to 92% (*P* < .006).

**Conclusions:**

Implementing evidence-based interventions in a learning network in East Africa improved compliance with IPC standards and occupational health competencies, which are critical to protecting HCWs and preventing pathogen transmission in healthcare facilities. This learning network approach can serve as a model for other regions or be implemented to address other public health emergencies.

## Teaser

A regional infection prevention and control (IPC) learning network was implemented in 20 tertiary care hospitals in East Africa. Evidence-based interventions, including routine assessments, trainings, and a collaborative quality-improvement project, improved compliance with IPC standards and occupational health competencies.

The global burden of healthcare-associated infections (HAIs) remains a significant challenge, particularly in low- and middle-income countries (LMICs), where HAI prevalence can reach up to 15% of hospitalized patients [1]. These infections not only compromise patient safety but also contribute to increased morbidity, mortality, and healthcare costs. The coronavirus disease 2019 (COVID-19) pandemic further exacerbated these challenges, overwhelming healthcare systems and exposing systemic gaps in infection prevention and control (IPC) practices [2]. Both the Ebola outbreaks and the COVID-19 pandemic demonstrated that healthcare worker (HCW) morbidity and mortality affect the capacity for crisis management and create challenges in health services [2]. During the COVID-19 pandemic, the prevalence of infection was disproportionately higher among HCWs than in the general population. Although HCWs represent less than 3% of the population in most countries and less than 2% in almost all LMICs, approximately 14% of COVID-19 cases reported to the World Health Organization (WHO) were among HCWs. Improving personal protective equipment (PPE) availability and appropriate use, screening of HCWs, and capacity building in IPC practices among HCWs are key strategic approaches to protecting HCWs during outbreaks [3–5].

The COVID-19 pandemic necessitated measures to protect HCWs, particularly those on the frontline [4], and to maintain continuity of essential healthcare services [4, 6]. The role of IPC during the pandemic was fundamental to controlling transmission within healthcare settings through developing guidelines and standard operating practices (SOPs), training, supporting decision-making processes, and promoting safe services to minimize the risk of infection among HCWs, visitors, and patients [7–11].

Learning networks and communities of practice have been identified as effective platforms for rapidly sharing resources and best practices to respond to health systems challenges [5, 6]. Through a learning network setting, IPC stakeholders (eg, frontline HCWs, IPC focal points, facility managers, program managers, and policymakers) have the opportunity to harness the benefits of connecting with colleagues across varied geographic settings to respond to specific challenges related to HCW protection. During the COVID-19 pandemic, there was an urgent need to rapidly improve compliance with IPC standards to protect HCWs, patients, and visitors. Healthcare workers are a critical resource in all health systems, and thus, protecting HCWs by improving IPC knowledge, including the appropriate use of PPE, through a learning network would be a practical strategy to ensure HCW safety [6].

International Center for AIDS Care and Treatment Program, Columbia University (ICAP) at Columbia University, in collaboration with the Ministries of Health (MOHs) in Ethiopia, Kenya, Tanzania, and Uganda and the US Centers for Disease Control and Prevention (CDC), designed and implemented an IPC learning network that brought together IPC professionals across neighboring countries in East Africa to collaborate, exchange best practices, and share innovation in IPC. The project aimed to improve compliance with IPC standards in hospitals and IPC focal point competencies by implementing an IPC learning network.

## METHODS

The East Africa IPC Learning Network (EAILN) was launched in 2020 in 4 countries. In collaboration with the MOHs, 20 tertiary care hospitals (5 in Ethiopia, 6 in Kenya, 5 in Tanzania, and 4 in Uganda) were identified to enroll in the EAILN using selection criteria. Selection criteria included a high volume of patients, presence of an IPC focal point with dedicated time for IPC work, and proximity between hospitals. The EAILN was designed and implemented to build capacity and a system for the improvement in IPC and occupational health to prevent transmission of pathogens to HCWs, patients, and visitors.

The following activities were implemented as part of the EAILN to identify and address gaps and measure improvement: (1) baseline and quarterly assessments of hospital compliance with IPC standards to identify gaps and measure progress; (2) baseline and annual assessments of self-reported IPC focal point competencies to identify gaps in IPC knowledge and practices, inform individual professional development plans, and measure progress; (3) weekly virtual case-based learning sessions and in-person supportive supervision visits by trained mentors; and (4) implementation of a collaborative quality improvement (QI) project on the use of PPE.

### Hospital IPC Compliance Assessments

Baseline and quarterly assessments of hospital IPC systems and practices were performed during January 2021 through June 2023 by the facility team using a tool adapted from the US CDC's facility readiness assessment for COVID-19: IPC considerations in non-US healthcare settings [12]. The assessment tool had 90 questions that covered 10 IPC domains, with 6 specific to compliance with IPC standards: (1) training, (2) patient and visitor flow, (3) triage and screening (eg, Ebola, COVID-19), (4) visitor management, (5) COVID-19 patient care, and (6) screening HCWs for respiratory diseases including COVID-19. Data were collected on the remaining 4 IPC domains (coordination, guidelines and SOPs, communication and reporting, supplies and infrastructure) but are not presented here. The assessment tool used response options of “yes” and “no” for each question. A response of “yes” for a given criterion indicated that specific requirements had been fulfilled. For example, an item under the domain of screening HCWs for respiratory diseases including COVID-19 was, “Facility has screening system in place for all HCWs prior to beginning work and/or entering the facility.” Summary scores were calculated for each domain as proportions with the sum of “yes” responses in the numerator and the total number of “yes” and “no” responses in the denominator. Results from the quarterly hospital IPC assessments informed the weekly case-based learning sessions, continuous mentorship, and sharing of relevant resources and tools to address identified gaps.

### IPC Focal Point Competency Assessments

Baseline and annual assessments of IPC focal point competency were self-administered using an electronic questionnaire that asked IPC focal points to rate their proficiency in 9 IPC core competencies, including occupational health. The IPC focal points were sent a secure link via an online platform to collect the data.

Data presented here are limited to the occupational health IPC competency, which includes the following components: (1) assess risk of occupational exposure to infectious diseases (eg, *Mycobacterium tuberculosis*, bloodborne pathogens); (2) describe how a staff member with an infection can pose a risk to other HCWs, patients, or visitors; (3) describe work practices that reduce the common risk of occupational exposure; (4) understand methods to handle blood and body fluids to prevent exposure safely; (5) articulate the process for reporting blood/body fluid exposure in the workplace; (6) describe postexposure management tasks to occupational injury or body fluid; and (7) collaborate with occupational health to establish a screening, postexposure management, and immunization program for HCWs.

Focal points were asked to rate their competency using a Likert scale of 1 to 5 for each component. Scores were derived from responses using the following scale: (1) I am not familiar with this competency, (2) I have some understanding but need more practice and support, (3) I can do this with supervision, (4) I can do this independently, and (5) I can teach others. Median scores of each component of the occupational health IPC competency domain were calculated, and results were used to identify gaps in IPC focal point capacity and measure progress over time, create individual professional development plans, and prioritize topics for case-based learning sessions and one-on-one mentorship for improvement.

### Weekly and Ongoing Education

Virtual weekly learning sessions updated participants on recommended IPC standards and practices using a case-based approach, shared guidance on using resources and tools for improvement, and provided participants with an opportunity to share experiences and lessons learned. The ICAP IPC technical experts also conducted routine virtual follow-up and feedback and quarterly in-person supportive supervision and mentorship visits to participating hospitals, and facilitated facility-to-facility exchange visits within the participating countries to foster collaboration and exchange of best practices.

### QI Collaborative

A 7-week virtual QI training was provided (via the weekly learning sessions) to MOHs and facility IPC teams to build capacity in designing and implementing QI projects. The training included weekly sessions and consecutive teamwork assignments to practically use and complete QI methods and tools. Following the training, all participating hospitals implemented a QI project to improve the appropriate use of PPE. Use of the same aim statement and monitoring indicators enabled participants to collaborate and share best practices.

At the start of the collaboration, participating hospitals conducted root cause analyses, identified contextually appropriate change ideas, selected target wards, collected and reviewed baseline data, developed aim statements and monitoring indicators, and presented their projects to facility leadership and staff. Each hospital team used a 25-question checklist developed to assess PPE topics, including the presence of SOPs and training, availability and storage, donning and doffing, and appropriate use. While implementing the QI projects, hospital QI teams collected monthly data by observing PPE use among staff, received supportive supervision and mentorship from ICAP technical experts, conducted interactive tests of change ideas (interventions) using the Model for Improvement and its Plan-Do-Study-Act (PDSA) cycle, tracked performance using indicators, analyzed QI run charts, and shared results and exchanged best practices during learning sessions [13].

Interventions that were implemented included the following: distribution of SOPs and job aids, on-the-job training and orientation, supervision and mentorship to the ward in-charge and HCWs, integration of IPC practices (including appropriate use of PPE) in ward daily spot checks and supervision, frequent monitoring of minimum PPE stock level, providing adequate PPE supplies, frequent audits and feedback, labeling of isolation rooms with instruction on PPE requirements, and engaging key persons from different units to monitor PPE use and provide timely feedback.

Upon completion of the QI collaborative, each hospital team presented their results and shared a report with their respective MOH to scale-up best practices and successful change ideas to other facilities in the country.

### Ethical Considerations

This project was certified by Columbia University to meet therigor of its data protection and data security standards. The protocol was approved as non–human subjects research determination by the Institutional Review Boards (IRBs) at Columbia University (AAAT7684, Y01M00). The project received IRB approval from the Uganda Virus Research Institute (GC/128/03/001), the Kenya Medical Research Institute (RES 7/3/1 #4741), the Ethiopia Public Health Association (EPHA/0G/912/22), and the National Institute for Medical Research, Tanzania (NIMR/HQ/R.8a/Vol.IX/4466). This activity was reviewed by the US CDC, deemed not research, and was conducted consistent with applicable federal law and US CDC policy including 45 CFR part 46 (1991); 21 CFR part 56 (1981); 42 USC §241[d] (1944); 5 USC §552a (1974); 44 USC §3501 et seq (1980).

### Data Analysis

Quantitative data were collected electronically and uploaded to a cloud-based system (SurveyCTO); no personally identifying information was collected [1, 14]. Aggregate anonymous data were used to generate descriptive statistics and summaries for the hospital IPC assessments, IPC focal point competency assessments, and QI data. Statistical analyses were performed using Microsoft Excel and SAS software; a Z-test for proportions was used to assess significant changes in compliance with appropriate PPE use.

## RESULTS

### Hospital IPC Compliance Assessments

**Figure 1.**
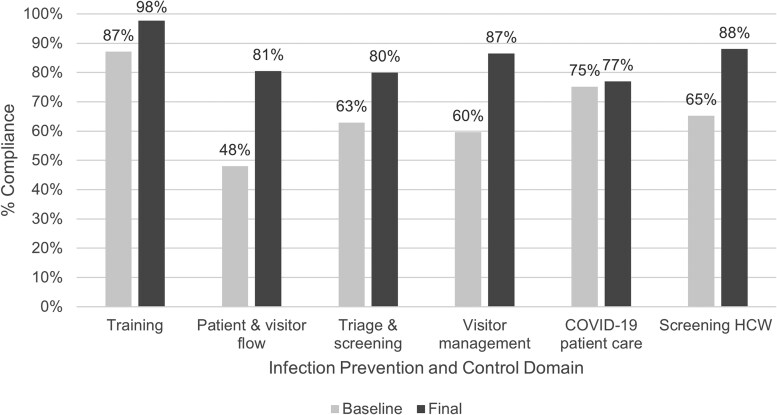
Aggregate compliance with IPC domains in 20 hospitals in Ethiopia, Kenya, Tanzania, and Uganda at baseline (Q1, 2021) and final (Q2, 2023) assessments. Abbreviations: COVID-19, coronavirus disease 2019; HCW, healthcare workers; IPC, infection prevention and control; Q, quarter.

**Figure 2.**
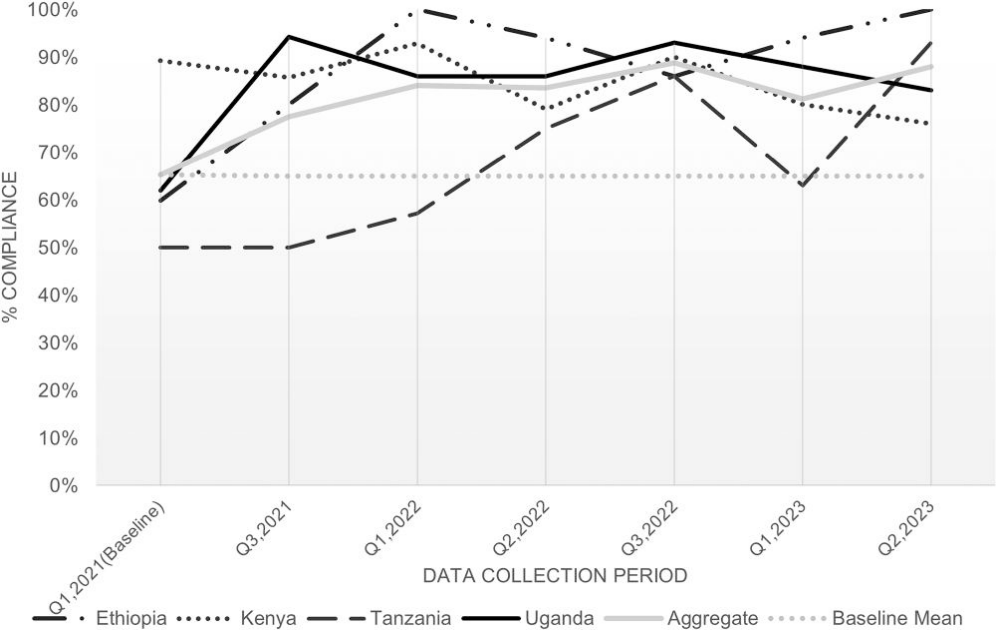
Compliance with screening of healthcare workers for respiratory diseases including COVID-19, by country: Q1 (2021)–Q2 (2023). Abbreviations: COVID-19, coronavirus disease 2019; Q, quarter.

Results from the hospital IPC compliance assessments demonstrated increased compliance across all 6 domains when comparing baseline and final assessments (aggregated results from all participating hospitals are shown in Figure 1). The largest improvements were in patients and visitor flow (from 48% to 81%), visitor management (from 60% to 87%), and HCW screening for respiratory diseases including COVID-19 (from 65% to 88%). Compliance with HCW screening for respiratory diseases improved in Ethiopia (+40%), Tanzania (+43%), and Uganda (+21%), but declined in Kenya (−24%) over the project period (Figure 2).

### IPC Focal Point Competency Assessments

**Figure 3.**
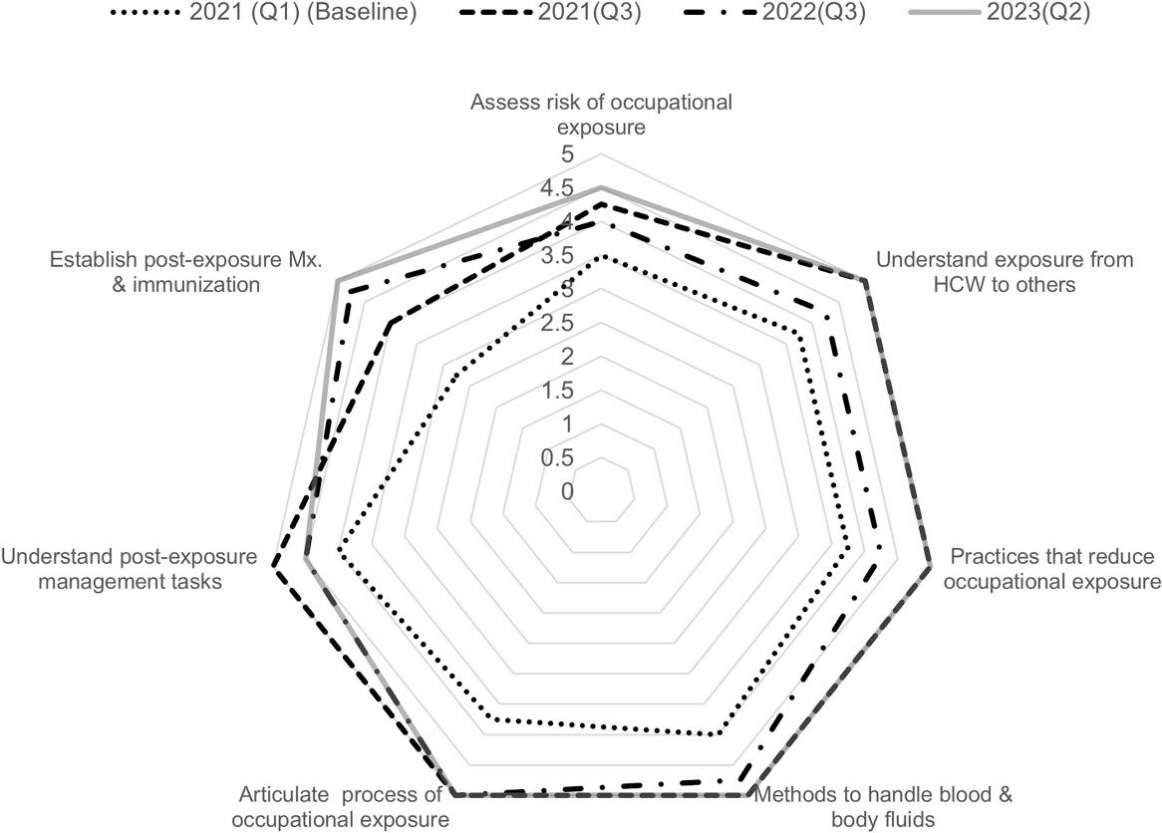
Aggregate infection prevention and control focal point competency assessments on occupational health: Q1 (2021)–Q2 (2023). Abbreviations: HCW, healthcare workers; Mx, management; Q, quarter.

Results from baseline IPC focal point competency assessments on occupational health showed that there were gaps in knowledge and practice for all components (aggregated results from all participating hospitals are shown in Figure 3). Baseline scores were lowest for assessment of the risk of occupational exposure to infectious diseases (median score of 3.5) and establishment of a screening, postexposure management, and immunization program for HCWs (median score of 2.75). The final assessment showed that there was improvement in all components of the occupational health competency, all with aggregate median scores of 4.5 or higher.

### QI Collaborative

**Figure 4.**
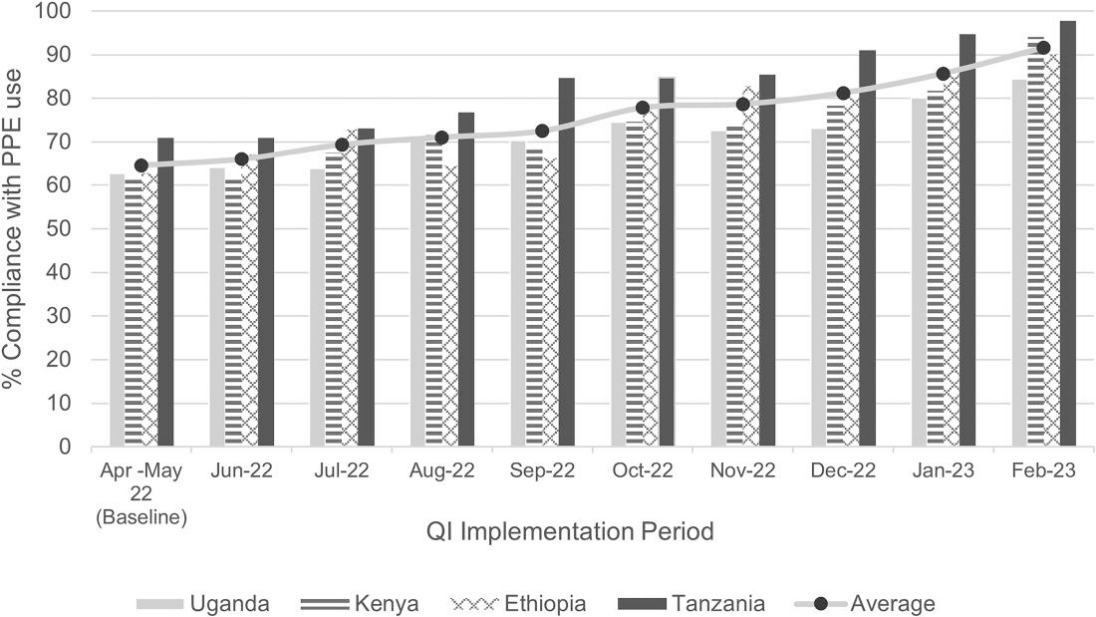
Aggregate average compliance with appropriate use of PPE, overall and by country, during the 9 months of QI collaborative implementation. Abbreviations: PPE, personal protective equipment; QI, quality improvement.

The QI collaborative was implemented as part of the EAILN from April 2022 through February 2023. Eighteen of the 20 hospitals completed the QI collaborative (4 in Kenya, 5 in Ethiopia, 5 in Tanzania, and 4 in Uganda). The average compliance with appropriate use of PPE (aggregated across all hospitals) showed a significant increase, from 65% at baseline to 92% at the end of the QI collaborative (*P* < .006) (Figure 4). There was variation in compliance with appropriate use of PPE across countries. However, increases in compliance across the implementation period were noted for hospitals in all countries, with absolute increases in percentage points of 27 in Ethiopia and Tanzania, 32 in Kenya, and 22 in Uganda.

## DISCUSSION

This paper describes a community of practice using a regional approach to protect HCWs during a public health emergency. The EAILN demonstrated substantial improvements in compliance with IPC practices that protect HCWs through a combination of facility IPC assessments, IPC focal point competency assessments, regular training and learning sessions, and a QI collaborative on PPE use. The strength of the project included participation of multiple facilities across 4 LMICs in East Africa and the variety of interventions to support change. The success of the EAILN is consistent with findings from a recent review of the effectiveness of IPC interventions in healthcare facilities in Africa, in which authors highlighted the importance of continuous IPC education and training and implementation of multimodal IPC interventions [15].

Routine assessments of hospital IPC compliance were used to identify gaps and monitor progress over time, as interventions were being implemented. Overall, compliance with screening of HCWs for respiratory infections improved in hospitals in Ethiopia, Tanzania, and Uganda during the project; however, overall compliance in Kenya decreased during the study period. The decline in Kenya might have been related to changes in government and facility leadership resulting in further disruption of the implementation of IPC policies. Also, staff reshuffling likely led to high staff turnover, which could result in inconsistent messaging and enforcement of protocols. It is also possible that facility leadership and IPC staff underestimated the need for continued strict measures when rates of COVID-19 declined, resulting in the relaxing of some IPC efforts. Sustained IPC efforts, consistent training, and the establishment of stable, long-term policies resilient to political and staffing changes are essential to prevent complacency and ensure future preparedness against health threats [7, 8]. Regular screening of HCWs for respiratory diseases and other potential infectious pathogens reduces the risk of pathogen transmission in healthcare settings, protects HCWs, and allows healthcare facilities to continue to provide care for patients [7–9]. A study conducted in the Philippines demonstrated that early initiation of COVID-19 screening for HCWs reduced the risk of infection and transmission in their healthcare facilities [11]. In this project, compliance with HCW screening varied over the duration of the project period and by country. It is possible that these findings are, in part, related to changes in the epidemiology of the COVID-19 pandemic and that compliance decreased when there was lower perceived infection risk. Despite its importance, screening of HCWs is not widely practiced in many countries, especially in LMICs [10, 11, 16].

Strengthening evidence-based knowledge and practices among IPC focal points is fundamental for HCW safety and ensuring high-quality, safe, and resilient healthcare services. Annual assessments of IPC focal point competencies were used to generate tailored professional development plans and to guide EAILN interventions, including topics covered during case-based learning sessions, supportive supervision and mentorship visits, and training opportunities. This approach resulted in overall improved scores on all components of the occupational health competency, suggesting increased knowledge and capacity in those areas. Previous studies have also demonstrated the importance of prioritizing educational needs based on HCW competency in IPC practices and by examining their current performance and perceived importance of improving their IPC knowledge and practices [16, 17].

Compliance with the appropriate use of PPE among HCWs was initially low (average of 65%) across EAILN facilities and showed significant improvement (average of 92%) following the QI collaborative. Studies have demonstrated that inadequate compliance with guidelines on the use of PPE and shortages of PPE are critical challenges in LMICs [5, 17]. A study conducted in various African countries during the COVID-19 pandemic highlighted that HCWs in only 61% of healthcare facilities wore all recommended PPE [3]. Cheng et al [3] demonstrated that appropriate use of PPE (including N95 respirator, gown, gloves, and goggles [or face shield]) by HCWs was associated with no cases of COVID-19 over a 42-day observation period. The appropriate use of PPE by HCWs is a standard safety protocol; ensuring the availability and appropriate use of PPE among HCWs is essential for protection against exposure to pathogens and reducing transmission of infectious pathogens within healthcare facilities. Several studies have demonstrated that adequate training on PPE donning and doffing, appropriate use, and continuous supply of adequate PPE minimize the risk of infection among HCWs when providing healthcare services, especially during outbreaks such as COVID-19 [4–6]. In the EAILN, multiple interventions were tested during the QI collaborative and successful interventions included continuous learning sessions and education, frequent audits and feedback, and monitoring PPE stock level and providing adequate PPE supplies. Implementation of those interventions resulted in substantive improvements in compliance with PPE use (from an average of 65% to 92%). Other learning networks could use a similar approach to improve IPC practices.

Three key limitations of this project should be considered. First, the participating facilities were selected by the MOHs based on their status as high-performing hospitals with existing IPC programs and focal points. As such, the results might not be replicable in other facilities or settings, particularly those that lack functional IPC programs. Second, the IPC improvements heavily relied on interventions that included mentorship and supervision supported by external partners, which may not be available in other settings. Finally, the EAILN's achievements occurred within the broader context of multiple efforts to strengthen IPC during the COVID-19 pandemic, including initiatives aimed at preventing pathogen transmission and protecting HCWs. These additional efforts likely contributed to the observed improvements, making it difficult to attribute results solely to this project. Future efforts could address these limitations by adapting the EAILN model to diverse settings, ensuring sustainability through local capacity building, and considering context-specific factors that may influence replicability.

The EAILN demonstrated that a multimodal and regional learning network approach improved professional development through competency-based IPC education and contributed to the success of a QI project in improving compliance with IPC practices. The model's success in increasing compliance with IPC measures highlights the potential for the integration of a learning community of practices into health systems to sustain achievements and scale-up improvements across other facilities. For example, the 20 EAILN hospitals have the potential to share best practices and provide mentorship to neighboring healthcare facilities, serving as hubs for scaling up and disseminating effective IPC strategies. Establishing similar networks across other regions or in response to future public health emergencies could replicate these successes.

The EAILN approach serves as a model for LMICs, demonstrating how learning networks can implement multimodal strategies to protect patients and HCWs while addressing public health challenges. Its success was driven by close collaboration with the MOH, tailored mentorship and supervision, and the integration of competency-based IPC education into HCW training programs. Key sustainability strategies included the following: (1) establishing national IPC Communities of Practice (CoPs) under MOH oversight and (2) institutionalizing IPC policies and practices within facility IPC programs. At both national and regional levels, leveraging networks like the EAILN can enhance coordination and resource sharing, facilitate technical support, and promote cross-border knowledge exchange. This model provides a scalable framework for implementing multimodal IPC strategies and strengthening preparedness for future public health emergencies.

Globally, the EAILN model could inform pandemic preparedness by demonstrating the value of collaborative networks in rapidly scaling up IPC interventions, particularly in resource-limited settings. Additionally, its principles can be integrated into routine healthcare safety policies to promote resilience among HCWs, reduce HAIs, and improve patient outcomes. Governments, policymakers, and international organizations should consider adapting this approach to strengthen global health systems, ensuring that HCW safety remains a central pillar of pandemic preparedness and routine healthcare delivery.

## Notes

***Author contributions***. G. K., M. R., E. B., L. B., C. D., E. K., M. P., I. K., and C. H. contributed to conceptualization; G. K., I. O., and R. M. contributed to data curation; G. K. and R. M. contributed to formal analysis; G. K., M. R., E. B., and C. H. contributed to funding acquisition; G. K., I. O., and R. M. contributed to investigation; G. K., I. O., M. R., E. B., L. B., E. K., M. P., I. K., and C. H. contributed to the methodology; G. K., I. O., M. R., E. B., L. B., C. D., and C. H. contributed to project administration; G. K. and L. B. contributed to resources; R. M. contributed to software; G. K., I. O., M. R., E. B., C. D., J. H., and C. H. contributed to supervision; G. K. and M. R. contributed to validation; G. K. and R. M. contributed to visualization; G. K. contributed to writing the original draft of the manuscript; and I. O., M. R., E. B., L. B., E. K., M. P., J. H., I. K., and C. H. contributed to reviewing and editing the manuscript. All authors have read and approved the final manuscript.

***Acknowledgments***. The authors acknowledge those who led and monitored the project implementation, including the respective Ministries of Health, CDC country offices, ICAP country offices, facility managers, IPC focal points, and ICAP IPC mentors in the 4 countries. They also acknowledge Christine Mburu, Mark Hawken, Sam Biraro, Haruka Maruyama, Zenebe Yirsaw, Doris Odera, Berhanu Tekle, John Kahemele, Paul Katongole, Linus Ndegwa, Beniam Feleke, Alemu Dersaw, Redempta Mutei, George Nyamizi, Kelvin Tito, Nicholas Sewe, Mariam Mmanywa, and Isabel Redman who were engaged and supported the implementation of the project. They thank Emily Peterson for her thoughtful review of the manuscript.

***Financial support.*** This project was supported by Cooperative Agreements NU2GGH002171 and NU2GGH002216 from the US Centers for Disease Control and Prevention.
